# Acknowledgment of Invited Editors

**DOI:** 10.1128/mBio.00731-18

**Published:** 2018-04-24

**Authors:** Arturo Casadevall

## EDITORIAL

On behalf of the editors of *mBio*, I gratefully acknowledge the following individuals, who served as invited editors for the journal in 2017. While not members of the Board of Editors, invited editors serve an important role in the review process. Invited editors are experts in their fields of research who add an additional level of quality to the review process. An editor may assign a paper to an invited editor when he/she would like to have an additional expert opinion of the reviews or when the subject area falls outside the editor’s primary area of expertise.

The time and effort of the following experts in handling articles have been essential to ensuring the high quality of our publications, and their help is greatly appreciated.

Dan I. Andersson
Martin Fabian Bachmann
Shweta Bansal
Fernando Baquero
Christopher F. Basler
Joseph Bondy-Denomy
Arpita Bose
Michael J. Brennan
Joseph Bresee
Roland Brosch
C. Titus Brown
Rut Carballido-López
Byron Caughey
Jean Celli
Swaine L. Chen
Peter Chien
Jeff A. Cole
Vaughn S. Cooper
Bryan R. Cullen
Christina A. Cuomo
Andrew J. Darwin
Rajendar Deora
Floyd E. Dewhirst
Alain Filloux
Klas Flardh
Kevin Foster
Vance G. Fowler
Philippe Gerard
Deanna Gibson
N. Louise Glass
FIlipa Godoy-Vitorino
Vernita Gordon
Jeffrey A. Gralnick
Jean Gruenberg
Karen Guillemin
John S. Gunn
David L. Gutnick
Lynn E. Hancock
Nancy D. Hanson
Robert L. Harrison
Jeffrey P. Henderson
Betsy Herold
Kelly T. Hughes
David A. Hunstad
Alexander Hüttenhofer
Michael Ibba
Akiko Iwasaki
Jorgen Johansson
Andreas Kappler
Krystyna M. Kazmierczak
Corby Kistler
David M. Knipe
Laura J. Knoll
Arash Komeili
Richard J. Kuhn
Mamuka Kvaratskhelia
Nathaniel Roy Landau
Stanley M. Lemon
Kim Lewis
Douglas R. Lowy
Rebecca Marie Lynch
Andrew J. Macpherson
William Margolin
Eric Martens
Rachel M. McLoughlin
Dennis W. Metzger
Joachim Morschhäuser
Joseph Mougous
Christian Munz
Kevin Myles
Julie Overbaugh
Andreas Peschel
Everett C. Pesci
Thomas Pietschmann
Ann M. Powers
Richard A. Proctor
Matthew Mark Ramsey
Timothy D. Read
Anthony R. Richardson
Gloria Marcela Rodriguez
Forest Rohwer
Antonis Rokas
Tony Romeo
Susan M. Rosenberg
Philip C. Rosenstiel
Gian Maria Rossolini
Monica J. Roth
Craig R. Roy
Ruth M. Ruprecht
Michael J. Sadowsky
John-Demian Sauer
Christa M. Schleper
Viviana Simon
Abraham L. Sonenshein
Justin Sonnenburg
Manjula Sritharan
Gurol Suel
Nobuhiro Suzuki
Daniel J. Thornhill
Raphael Valdivia
Susana T. Valente
Marjan W. van der Woude
Stefanie N. Vogel
Mark J. Walker
Jens Walter
David S. Weiss
Roy D. Welch
Angus C. Wilson
Alan J. Wolfe
Otto O. Yang
Fitnat H. Yildiz
Vincent B. Young
Jae-Hyuk Yu
Gongyi Zhang
Xinning Zhang

